# Genomic landscape of biliary tract cancer and corresponding targeted treatment strategies

**DOI:** 10.1007/s10147-025-02761-x

**Published:** 2025-04-25

**Authors:** Daisaku Yamada, Shogo Kobayashi, Yuichiro Doki, Hidetoshi Eguchi

**Affiliations:** Department of Gastroenterological Surgery, Graduate School of Medicine, Osaka University, 2-2E2, Yamadaoka, Suita City, Osaka 565-0871 Japan

**Keywords:** Biliary tract cancer, Cancer genome testing, Target therapy

## Abstract

Biliary tract cancers (BTCs) are classified on the basis of their anatomical origin, and the feasibility of surgical resection depends on the tumor location and extent of progression. However, for unresectable BTCs, systemic therapy has been uniformly applied. Gemcitabine and cisplatin (GC) therapy and GC-based therapies were established as the first-line standard BTC treatment. However, no highly effective second-line therapy has been established, and the prognosis remains poor, highlighting the need for further therapeutic advancements. Meanwhile, the era of precision medicine has expanded the use of genetic testing, leading to the identification of actionable molecular targets in BTC. Several targeted therapies, including FGFR inhibitors and IDH1 inhibitors, have been developed, offering new second-line treatment options and the potential for first-line use in appropriate cases. Notably, the frequency of these genetic alterations varies depending on the tumor location, demonstrating the molecular heterogeneity of BTC. Therefore, it has been recognized that a tailored treatment approach for each BTC patient may be more effective than uniform systemic therapy. Consequently, although routine genetic testing before initiating systemic treatment is currently limited by the medical environment (e.g., cost, accessibility, regional differences), it is recommended in ESMO guideline and might be increasingly advocated. However, BTC harbors a wide range of genetic alterations, and numerous targeted therapies are being developed accordingly. This review provides an overview of the reported genetic alterations in BTC, the frequencies of these alterations, and the corresponding targeted therapies, emphasizing the evolving role of precision medicine in BTC treatment.

## Introduction

Biliary tract cancer (BTC) arises from cholangiocytes located along the biliary tree, from the intrahepatic ducts to the ampulla of Vater. Classifications of BTC are highly diverse. On the basis of its anatomical origin, BTC can be categorized as intrahepatic cholangiocarcinoma (iCCA) or extrahepatic cholangiocarcinoma (eCCA). eCCA can be further subclassified into perihilar cholangiocarcinoma (pCCA), distal cholangiocarcinoma (dCCA), or gallbladder cancer (GBC). Additionally, ampulla of Vater carcinoma (AVC) is considered distinct subclass and may be classified as a biliary, pancreatic, or duodenal cancer on the basis of its cellular origin. While AVC is included as a subset of BTC according to some classifications [1], it is excluded in other classifications [2]. Although BTC is a rare malignancy, its incidence is increasing worldwide [3].

Historically, the classification of BTC has been aligned with therapeutic objectives. Anatomical classification is useful for assessing the extent of tumor progression and determining the appropriate surgical approach for treatment. For example, BTCs requiring hepatic resection, extrahepatic bile duct resection, or pancreaticoduodenectomy have been grouped accordingly. However, for unresectable BTCs, systemic chemotherapy has been applied universally, treating BTCs as a single entity originating from cholangiocytes. The landmark ABC-02 trial in 2010 established gemcitabine and cisplatin (GC) therapy as the standard BTC treatment [4]. GC therapy is effective for advanced BTC, and several novel therapies based on GC therapy have been tested [5, 6], with some demonstrating superior efficacy [7–9]. However, no second-line treatment has been established with sufficient efficacy, and the median overall survival for advanced BTC remains less than one year in most reports [10].

Recent advancements in genomic analysis have shed light on the genetic alterations underlying BTC [11–24]. In particular, iCCA has emerged as a focus of genomic research because of the feasibility of obtaining adequate tumor samples for analysis [25]. Studies have revealed actionable mutations such as FGFR and IDH1/2 alterations, leading to the development of targeted therapies that have progressed through phase II/III trials [26–29] and are now widely used in clinical practice (Table 1) [26–35]. These successes have spurred further genomic investigations for other BTC subtypes, resulting in the identification of additional actionable mutations and the ongoing development of novel targeted therapies (Tables 1, 2).

**Table 1:** Frequencies of gene mutations and target drugs

	iCCA		eCCA				
Gene mutation	Rank	Frequency	Rank	Frequency	Valid target drugs (FDA-approved)	Phase	Outcomes
*KRAS*	**#1**	7–54%	**#2**	37–57%	None		
*TP53*	**#2**	18–27%	**#1**	18–68%	None		
*ARID1A*	**#3**	18–23%	**#5**	14%	None		
*IDH1/2*	**#4**	7–30%	**#10**	0–5%	Ivosidenib	III	Prolonged PFS, 2.7 vs. 1.4 months
*CDKN2A/B*	**#5**	9–27%	**#3**	9–28%	None		
*EGFR*	**#6**	8–27%	**#9**	1%	None (Failure)		
*FGFR2*	**#7**	5–16%	**#11**	0–4%	Pemigatinib Futibatinib	II II	ORR 35.5%, PFS 6.9 months ORR 41.7%, PFS 9 months
*PI3K*	**#8**	7%	**#7**	5%	None		
*ERBB2*	**#9**	3–8%	**#4**	1–27%	Zanidatamab	IIb	ORR 41.3%, PFS 5.5 months
*BRAF*^*V600E*^	**#10**	1–5%	**#12**	< 1%	Dabrafenib + trametinib	II	ORR 53% PFS 9.0 months
*BRCA1/2*	**#11**	0–3%	**#8**	2–3%	Standard chemotherapy (GC regimen)		
*RET*	**#12**	0–6%	-	-	None		
*NTRK*	**#13**	< 1%	**#13**	< 1%	Entrectinib* Larotrectinib*	I/II I/II	ORR 100%**, PFS 9.3** months ORR 50%†, PFS 7.3†months
*SMAD4*	**–**	–	**#6**	11–25%	None		

*iCCA* intrahepatic cholangiocarcinoma, *eCCA* extrahepatic cholangiocarcinoma, *FDA* U.S. Food & Drug Administration, *PFS* progression-free survival, *ORR* objective response rate, *GC* gemcitabine and cisplatin

^*^Solid tumor

^**^Only one BTC case was included

^†^Two cholangiocarcinoma cases were included

**Table 2:** Possible targeted drugs for gene mutations

Target	Objectives	Clinical trial identifier	Phase	Possible targeted drugs	Outcomes
*KRAS*	Solid tumors	NCT05162443 NCT04185883 NCT05737706	II Ib I/II	Adagrasib (G12C) Sotorasib (G12C) MRTX1133 (G12D)	DCR: 100%(*n* = 8) Ongoing Ongoing
*TP53*	Solid tumors	NCT04383938	I/II	Eprenetapopt + pembrolizumab	BTC patients were not included
*ARID1A*	Solid tumors	NCT05023655	II	Tazemetostat	Ongoing
*IDH1/2*	Solid tumors	NCT03684811 NCT02273739	I/II I/II	Olutasidenib Enasidenib	ORR: 12.5% (*n* = 8) BTC patients were not included
*CDKN2A/B*	Solid tumors	NCT02693535 NCT02693535/NCT03297606 NCT04116541/NCT02187783	II II/ II II/ II	Abemaciclib Palbociclib Ribociclib	Ongoing Ongoing Ongoing Ongoing/ only one GBC patient included
*EGFR*	BTC	NCT04838964	II	MRG003	Ongoing
*FGFR2*	CCA	NCT02150967 NCT03773302 NCT01752920 NCT04087876	II III I/II II	Infigratinib Derazantinib	ORR: 23.1%(*n* = 108) Early termination ORR: 8.6% (*n* = 58) Ongoing
*PI3K*	Solid tumors	NCT06739395	II	Alpelisib	Ongoing
*ERBB2*	BTC Solid tumors CCA Solid tumors Solid tumors	NCT06467357 NCT05150691 NCT02999672 NCT06519110 NCT04579380	III I/IIa II II II	Trastuzumab-deruxtecan Trastuzumab + pertuzumab Trastuzumab-emtansine Neratinib Trastuzumab-tucatinib	Ongoing ORR: 23.1% (*n* = 39) ORR: 14.3% (*n* = 7*) Ongoing ORR: 46.7% (*n* = 30)
*BRAF*^*V600E*^	Solid tumors Malignancy	NCT02693535 NCT05768178	II II/III	Vemurafenib + cobimetinib	Only 2 BTC patients included Ongoing
*BRCA1/2*	BTC	NCT05222971	II	Olaparib	Ongoing
*RET*	Solid tumors Solid tumors	NCT03157128 NCT03037385	I/II I/II	Selpercainib Pralsetinib	Only one BTC patient included ORR: 66% (*n* = 3)
*SMAD4*	Solid tumors	NCT04116541	II	Regorafenib	Ongoing

*DCR* disease control rate, *BTC* biliary tract cancer, *GBC* gallbladder cancer, *ORR* objective response rate

^*^Included cases of pancreatic cancer

Interestingly, distinct genomic profiles among BTC anatomical classification subtypes have been revealed (Table 1). This finding might indicate that tumor location and the associated microenvironment play significant roles in carcinogenesis, even among cancers originating from cholangiocytes. Environmental factors also appear to influence BTC incidence, as evidenced by geographical and ethnic variations. For example, the incidence of BTC tends to be higher among Asians, particularly in Asian countries (incidence rate in Asia: 3–10/100,000 population; incidence rate in Europe or America: 1–3/100,000 population) [10, 36, 37].

Given the heterogeneity of BTC, the limitations of a one-size-fits-all therapeutic regimen have become apparent. Precision medicine, involving the selection of appropriate therapies tailored to the specific genetic and molecular characteristics of each patient's BTC, is increasingly recognized as essential [20, 38–43]. Actionable mutations are identified in one-third to one-half of BTC cases [44, 45]. In their BTC management guidelines, the European Society for Medical Oncology (ESMO) proposes a treatment strategy for advanced BTC in which genomic testing is performed upfront and targeted therapies are prioritized when actionable mutations are present [41]. In the absence of such mutations, systemic chemotherapy remains the standard of care [46]. The NCCN guideline also recommends performing genomic testing for BTC patients being considered for systemic chemotherapy [2].

Thus, genomic testing and targeted therapy are rapidly becoming central to the management of BTC. The aim of this review is to summarize the current status of genomic testing, the targeted therapies available in clinical practice, and promising future developments.

### Landscape of genomic alterations across BTC subtypes

To consolidate existing reports [12, 14, 16, 22], BTC is categorized into iCCA and eCCA in this review, and a summary of genomic mutation findings is provided. As shown in Table 1, some mutations are shared between subtypes, but the frequencies of these mutations differ significantly between iCCA and eCCA.

### iCCA

Genomic mutations in iCCA have been extensively reported, with the number of publications increasing yearly. The variety in mutation types and frequencies reported may reflect differences in epidemiology, treatment environments, and sample collection and preservation conditions across studies. However, there are also significant commonalities. Frequently reported mutations include those in KRAS, TP53, ARID1A, IDH1/2, CDKN2A/B, EGFR and FGFR. Less frequent mutations, such as those in PI3K, ERBB2 and BRAF, have also been reported. Furthermore, although rare, actionable mutations such as NTRK fusions and RET fusions are relatively well documented (Table 1).

### eCCA

There are fewer reports of genomic mutations in eCCA than in iCCA, but the number of such reports is increasing. Common mutations in eCCA include those in KRAS, TP53 and CDKN2A/B, whereas ERBB2 mutations, which are frequently reported in eCCA, occur at lower frequencies in iCCA. Both IDH1/2 mutations and FGFR mutations are rare in eCCA (Table 1).

### Genetic mutations frequently found in BTC

#### IDH1/2 (isocitrate dehydrogenase 1/2) mutations

IDH1 mutations are observed in 7–30% of BTCs, predominantly in iCCA (Table 1) [27, 44, 47–54]. IDH1/2 proteins normally participate in energy metabolism, producing α-ketoglutarate (αKG) from isocitrate with NADP+ as a coenzyme. However, mutant IDH1/2 proteins cannot produce αKG and instead produce D-2-hydroxyglutarate (D2HG) in an αKG-dependent manner, using NADPH as a coenzyme. This change in function inhibits αKG-dependent dioxygenases, disrupting the activity of related enzymes and affecting the hypoxia response and epigenetic regulation, thereby contributing to tumor progression. Recently, IDH1 inhibitors (e.g., ivosidenib) have been developed, and their efficacy in BTC was demonstrated in a phase III trial (ClarIDHy trial, Table 1) [26, 27]. Owing to their mechanism of action, IDH1 inhibitors can have broad impacts on tumors and differ fundamentally from cytotoxic anticancer agents, making further research into their use as first-line treatments or in combination with existing therapies highly promising [52, 54]. Another IDH1 inhibitor, olutasidenib, has been approved for the treatment of hematologic malignancies and has also shown efficacy in BTC (Table 2) [55]. For IDH2 mutations, enasidenib has been developed and tested in clinical trials for the treatment of solid tumors (Table 2). However, patients with BTC were not included in this trial, leaving room for further investigation. In the future, additional research into IDH-targeted therapies for BTC is highly anticipated.

#### FGFR (fibroblast growth factor receptor) mutations

FGFR mutations are observed in 5–16% of BTCs, primarily in iCCA (Table 1) [51–54, 56–59]. FGFR2 fusion proteins produced from these genes activate tyrosine kinases in a ligand-independent manner, leading to the activation of the FGFR pathway. FGFR pathway activation, in turn, activates downstream pathways, including the MAPK, PI3K-AKT, and STAT pathways, promoting antiapoptotic effects and cell proliferation. FGFR has five subunits (FGFR1–5), and several FGFR inhibitors (pemigatinib, an FGFR1–3 inhibitor; infigratinib, an FGFR1–5 inhibitor; futibatinib, an FGFR1–4 inhibitor; and derazantinib, an FGFR1-3 inhibitor) have been developed, with therapeutic efficacy demonstrated in phase II trials (Tables 1, 2) [28, 29, 60, 61]. Clinical trials have demonstrated the efficacy of these FGFR inhibitors as monotherapies in patients with FGFR mutations following standard treatment. Among these drugs, pemigatinib and futibatinib are approved for use in BTC by the FDA (U.S. Food & Drug Administration, https://www.fda.gov/). Currently, international phase III trials are ongoing to compare these drugs with GC treatment as first-line therapy (NCT03656536 [62], NCT03773302 [63], and NCT04093362). Further studies, such as studies in which synergistic effects with existing anticancer agents are evaluated, are highly anticipated.

#### ERBB2 (v-erb-b2 avian erythroblastic leukemia viral oncogene homolog 2) mutations

ERBB2 aberrations are identified in 3–8% of iCCA cases and 1–27% of eCCA cases (Table 1) [44, 52, 53, 64–67]. Cancer cells with ERBB2 aberrations overexpress HER2 (human epidermal growth factor receptor 2) receptors, increasing ligand sensitivity and activating downstream pathways (e.g., the MAPK and PI3K-AKT pathways). Molecular targeted therapies for BTC, such as trastuzumab, deruxtecan, pertuzumab, tucatinib and emtansine, which were initially developed for HER2-positive breast cancer, have been tested in phase II trials with favorable outcomes (Table 2) [68–71]. Among these, zanidatamab has been approved by the FDA for the treatment of HER2-positive BTC, demonstrating an objective response rate (ORR) of 41.3% and a progression-free survival (PFS) of 5.5 months in the HERIZON-BTC-01 trial [30]. Further clinical trials are ongoing (Table 2), and additional data are anticipated.

### Common genetic mutations in cancer

#### KRAS (Kirsten rat sarcoma viral oncogene homolog) mutations

Currently, there is no approved standard therapy targeting KRAS mutations in BTC. However, since KRAS mutations are relatively common in BTC (7–57%, Table 1) [44, 49, 51, 72–74], targeted therapy development is ongoing. Among KRAS mutations in BTC, the G12C mutation is rare (~ 1–2%), but KRAS G12C inhibitors, such as adagrasib and sotorasib, have already been approved by the FDA for the treatment of non-small cell lung cancer (NSCLC) and are now being investigated in clinical trials for the treatment of BTC (Table 2). Reports indicate promising treatment efficacy [75], raising expectations for expanded indications in the future. Other common KRAS mutations in BTC include G12D, G12V, and G13D. A small-molecule inhibitor targeting KRAS G12D, MRTX1133, is currently in clinical trials for the treatment of solid tumors, including BTC (NCT05737706). Further development is eagerly awaited.

#### TP53 mutations

Both iCCA and eCCA frequently exhibit TP53 mutations (Table 1) [44, 51, 72–74]. However, no actionable drug is currently available, as therapies directly targeting TP53 mutations are still in development. TP53 is a tumor suppressor gene, and TP53 mutations are highly prevalent across various cancer types. The diversity of TP53 mutations (missense mutations, deletions, and insertions) and the structural complexity of the p53 protein have made targeted therapy development challenging. While direct therapies are limited, indirect approaches are being explored. One promising strategy is the restoration of mutant p53 function via the use of small molecules. For example, APR-246 (eprenetapopt) was designed to stabilize mutant p53 structurally and restore its normal function [76]. The combination of eprenetapopt with immune checkpoint inhibitors is currently being investigated in clinical trials, potentially including BTC patients (Table 2) [77]. As research progresses, novel therapeutic strategies tailored to TP53-mutant cancers are anticipated.

#### ARID1A (AT-Rich Interactive Domain 1A) mutations

Both iCCA and eCCA frequently exhibit ARID1A mutations (Table 1) [44, 51, 72]. ARID1A is a component of the SWI/SNF (Switch/Sucrose Nonfermentable) chromatin remodeling complex and functions as a tumor suppressor gene. Mutations in ARID1A lead to DNA repair defects and transcriptional dysregulation, contributing to tumor progression. While no specific targeted therapies for ARID1A mutations are currently available, several indirect approaches are under investigation. PARP (ADP-ribose) polymerase) inhibitors and ATR (ataxia telangiectasia and Rad3-related) inhibitors, which target DNA repair deficiencies, may be effective [78]. Additionally, ARID1A mutations can increase the activity of EZH2 (Enhancer of Zeste Homolog 2), making EZH2 inhibitors (e.g., tazemetostat) promising therapeutic options [79]. These inhibitors are being evaluated in the treatment of BTC in clinical trials (Table 2, NCT05023655). Yoshino and colleagues demonstrated that ARID1A decreased histone H3K27 acetylation in cholangiocarcinoma cells, suggesting it as a potential therapeutic target [80]. Furthermore, tumors harboring ARID1A mutations may exhibit high microsatellite instability (MSI-H) or high tumor mutation burden (TMB), potentially rendering them responsive to immune checkpoint inhibitors such as pembrolizumab and nivolumab [81].

#### CDKN2A/B (cyclin-dependent kinase inhibitor 2A/B) mutations

Both iCCA and eCCA frequently exhibit CDKN2A/B mutations (Table 1) [44, 51, 72–74]; however, there is currently no direct targeted therapy for these mutations. CDKN2A is a tumor suppressor gene involved in cell cycle regulation and encodes the proteins p16 (INK4A) and p14 (ARF), which modulate the RB and p53 pathways. Loss of p16INK4A function due to CDKN2A mutations or deletions leads to increased CDK4/6 activity, uncontrolled cell cycle progression, and tumor growth. Consequently, CDK4/6 inhibitors (e.g., palbociclib, ribociclib, and abemaciclib) represent potential indirect therapeutic strategies. These inhibitors induce G1 cell cycle arrest and suppress tumor proliferation. CDK4/6 inhibitors for the treatment of BTC are currently being evaluated in clinical trials (Table 2, NCT02693535:TAPUR Group 4, Group 17, NCT04116541).

#### EGFR (epithelial growth factor receptor) mutations

EGFR mutations are frequently detected in iCCA (Table 1) [44, 82–85]. However, clinical trials of EGFR inhibitors (e.g., cetuximab, panitumumab, erlotinib) have not demonstrated sufficient efficacy [86–91], and no actionable drug is currently available. The efficacy of MRG003, an EGFR-targeting agent, for the treatment of EGFR-overexpressing BTC is being investigated in ongoing clinical trials (Table 2, NCT04838964). Future results are awaited with interest.

#### PI3K (phosphoinositide 3-kinase) mutations

Both iCCA and eCCA occasionally harbor PI3K mutations (Table 1) [44, 51]. Several targeted therapies for PI3K mutations are currently in development. PI3K mutations lead to dysregulation of the PI3K/AKT/mTOR signaling pathway, which promotes tumor growth and treatment resistance. This pathway is frequently altered in various cancers, and PI3K inhibitors, including the selective PI3K inhibitor alpelisib, which has been approved by the FDA for the treatment of PIK3CA-mutant HR-positive, HER2-negative breast cancer (based on the SOLAR-1 trial), have been developed [92]. Ongoing clinical trials are evaluating PI3K inhibitors in solid tumors, including BTC (Table 2, NCT06739395). Further advancements in treatment strategies are anticipated.

#### BRAF mutation (V600E)

BRAF mutations are observed across various cancer types, with the V600E mutation being particularly common in melanoma and colorectal cancer. In BTC, BRAF mutations are found in fewer than 5% of cases, with a higher prevalence in iCCA (Table 1) [52, 53]. The V600E mutation causes abnormal activation of the MAPK pathway independent of ligand stimulation, making it a therapeutic target for BRAF and MEK inhibitors. Clinical trials (ROAR trial) of dabrafenib (a BRAF inhibitor) and trametinib (a MEK inhibitor) in the treatment of BRAF V600E-mutated BTC have revealed promising results, and these drugs are approved by the FDA for use in the treatment of BTC (Table 1) [31, 93]. The combination of vemurafenib and cobimetinib is currently being investigated in ongoing trials (Table 2).

#### BRCA1/2 (breast cancer susceptibility gene) mutations

BRCA1/2 gene mutations are observed in 0–3% of BTC cases (Table 1) [94], making them relatively rare. BRCA genes are well known for their association with hereditary breast and ovarian cancers, and mutations in these genes lead to homologous recombination deficiency (HRD), resulting in the accumulation of DNA damage that drives carcinogenesis. In cancer cells harboring BRCA1/2 mutations, the inability to efficiently repair DNA interstrand crosslinks caused by platinum-based chemotherapy is expected to increase treatment efficacy. Since GC therapy is commonly used as the standard treatment for BTC [4, 7–9], platinum-based agents are often administered to all patients with advanced BTC regardless of BRCA1/2-mutation. Furthermore, the use of PARP inhibitors as second-line treatment is expected to prolong therapeutic effects, and clinical trials are currently ongoing [95].

#### RET (rearranged during transfection) mutation

The RET proto-oncogene encodes a transmembrane receptor tyrosine kinase, and RET mutations drive oncogenic transformation in various cancers [96]. In RET fusion mutations, the RET gene fuses with a partner gene, often encoding a coiled-coil domain, leading to ligand-independent constitutive dimerization and subsequent kinase activation [97, 98]. The RET inhibitors selpercatinib and pralsetinib have demonstrated therapeutic efficacy in patients with RET-mutated solid tumors (Table 2) [99, 100]. Although RET mutations are relatively rare in BTC (0–6%, Table 1) [101, 102], these clinical trials included a small number of BTC patients, suggesting that these agents may also be effective in BTC patients with RET mutations.

#### NTRK (neurotrophic tyrosine receptor kinase) mutations

Although NTRK fusions have been reported in only 0.2–0.7% of patients with BTC (Table 1) [103, 104], they are recognized as oncogenic driver genes in various tumor types. The NTRK gene family (NTRK1–3) encodes TRK proteins, which play crucial roles in normal neuronal development. However, when TRK fusion proteins are formed, they aberrantly activate tyrosine kinase activity, driving tumor progression. Two oral NTRK inhibitors, entrectinib and larotrectinib, are currently approved for use in patients with advanced NTRK fusion-positive solid tumors, including BTC. Both entrectinib and larotrectinib have demonstrated clinical benefits in phase I/II clinical trials [33, 105]. These agents represent promising treatment options for BTC patients with NTRK fusions.

#### SMAD4 mutation

There is currently no established specific targeted therapy for SMAD4 gene mutations. However, SMAD4 is a key molecule in the transforming growth factor-β (TGF-β) signaling pathway, and abnormalities in this pathway are observed in many cancer types. As a result, therapies targeting pathways indirectly affected by SMAD4 mutations are being investigated. Among the clinical trials of FDA-approved drugs, a part of the MegaMOST trial is evaluating the use of regorafenib in solid tumors with biallelic inactivation of SMAD4 (Table 2). SMAD4 mutations have been reported in 11–25% of patients with eCCA (Table 1) [49], and it is anticipated that some of these trials will lead to the development of a new actionable drug for BTC.

## Discussion

This review summarizes the current frequency of genomic mutations in CCA and their potential for targeted therapy. Interestingly, IDH1 mutations rarely overlap with cases harboring FGFR fusion genes [106]. In support of this observation, cluster analysis of next-generation sequencing (NGS) results has demonstrated that cases can be categorized into four groups on the basis of the presence of IDH mutations, FGFR mutations, TP53 mutations, and CDKN2A mutations [107]. If these findings commonly hold true, the cumulative frequency of these genetic abnormalities suggests that a significant proportion of BTC patients could benefit from one of these targeted treatments. Numerous clinical trials of those targeted therapies are ongoing (Table 2), and if favorable results are obtained, the proportion of actionable genomic mutations is expected to significantly increase in the near future, increasing hope for improved outcomes.

Current mainstream genomic testing relies on tumor biopsy samples analyzed via NGS. However, obtaining tissue biopsies of BTC, especially for eCCA [25], can be challenging in terms of safety and ensuring adequate tissue quality for comprehensive molecular testing [45, 108], with approximately 70% of samples yielding sufficient tumor content for analysis. To complement testing for patients whose samples are insufficient for analysis, blood-derived circulating tumor DNA (ctDNA) analysis has been utilized. The NCCN and ESMO guidelines recommend considering tissue-based genomic testing first, and if it is challenging, ctDNA testing should be considered [2, 41]. ctDNA analysis offers certain advantages over tissue DNA sequencing, being less invasive and increasingly available, with relatively high diagnostic accuracy for certain mutations (e.g., concordance rates: IDH1 mutation 87%; BRAFV600E 87%) [109]. However, lower concordance rates have been observed for certain mutations, such as FGFR2 fusions, which show an overall concordance rate of 18%, varying by fusion partner (FGFR2-BICC1 fusions 58%, FGFR2-other fusions 2.1%) [109]. For ctDNA, it is necessary to keep in mind the use of such tests with consideration for their limitations when conducting clinical practice, while this method also has the potential to enable real-time monitoring of genomic evolution. Further advancements in these methods are anticipated.

While promising, limitations of targeted therapy have also emerged. For example, the efficacy of gene-targeted treatments often diminishes after an average of 6–9 months (Table 1), possibly due to secondary mutations that confer resistance. ctDNA analysis revealed de novo point mutations in the FGFR2 gene, possibly conferring resistance to BGJ398 at the time of testing upon disease progression [110]. Goyal and colleagues reported the usefulness of ctDNA analysis for disease monitoring and the detection of acquired resistance during targeted therapy in a phase II study of the FGFR inhibitor infigratinib (BGJ398) [110]. Similar data were reported by Silverman and coworkers in the FIGHT-202 trial of pemigatinib in patients with FGFR-rearranged CCA by using either tissue or liquid biopsy testing [56]. Thus, in targeted therapy, not only for determining eligibility but also for predicting treatment efficacy, the use of blood samples to analyze ctDNA is expected to become increasingly valuable, highlighting the growing importance of this method.

Breakthroughs in targeted therapy have fundamentally different mechanisms of action compared with those of cytotoxic chemotherapies, potentially yielding superior efficacy. Moreover, the combination of targeted drugs with cytotoxic agents may produce synergistic, additive, or antagonistic effects, necessitating further exploration of optimal sequencing and safety. For example, the authors demonstrated a potential synergistic effect of FGFR inhibitors with gemcitabine [111]. Additionally, regarding FGFR2 inhibitors, the development of new, more potent selective FGFR inhibitors (e.g., RLY-4008, KIN-3248) is underway, which may circumvent drug resistance caused by point mutations, and there is growing anticipation for these advancements [112, 113]. Trials primarily focusing on targeted therapy alone are presented in Table 2; however, trials in which chemotherapy is combined with targeted therapy are also becoming more prominent. Future data are anticipated with great interest.

As targeted therapies demonstrate significant efficacy, the benefits and risks of their integration into treatment plans alongside surgical interventions must be carefully evaluated. Strategies such as initiating targeted therapy, followed by minimal surgical intervention and adjuvant cytotoxic chemotherapy (or immune therapy), may emerge as viable approaches.

This review presents the current landscape of BTC genomic alterations and corresponding targeted therapies. In the future, targeted therapy is expected to become central to treatment planning. With the growing complexity of treatment options, leveraging artificial intelligence (AI) for treatment decision-making is likely to become standard practice. However, robust data and systematic clinical trials are needed to train AI systems effectively. The authors hope that this review contributes to these advancements and the establishment of optimal treatment frameworks.
